# Clinical and Epidemiological Characteristics of *Streptococcus suis* Infections in Catalonia, Spain

**DOI:** 10.3389/fmed.2021.792233

**Published:** 2021-12-08

**Authors:** Javier Díez de los Ríos, Esteban Reynaga, Merce García-Gonzàlez, Jordi Càmara, Carmen Ardanuy, Jordi Cuquet, Maria D. Quesada, Marian Navarro, Anna Vilamala, Noemi Párraga-Niño, Sara Quero, Alba Romero, Rosa M. Benítez, Jacint Altimiras, Maria Luisa Pedro-Botet

**Affiliations:** ^1^Department of Internal Medicine, Hospital Universitari Vic, Barcelona, Spain; ^2^Department of Infectious Diseases, Hospital Universitari Germans Trias i Pujol, Badalona, Spain; ^3^Department of Microbiology, Hospital Universitari Arnau de Vilanova, Lleida, Spain; ^4^Department of Microbiology, Hospital Universitari Bellvitge-IDIBELL, Barcelona, Spain; ^5^Department of Internal Medicine, Hospital Universitari Granollers, Barcelona, Spain; ^6^Department of Microbiology, Hospital Universitari Germans Trias i Pujol, Badalona, Spain; ^7^Department of Microbiology, Hospital Universitari Vic, Barcelona, Spain; ^8^Infectious Diseases Unit, Fundació Institut d'Investigació Germans Trias i Pujol, Barcelona, Spain; ^9^CIBER de Enfermedades Respiratorias, CIBERES, Madrid, Spain; ^10^Institut d'Investigació i Innovació Parc Taulí, Sabadell, Spain; ^11^Department of Epidemiology, Hospital Universitari Vic, Barcelona, Spain; ^12^Department of Medicine, Universitat Autònoma de Barcelona, Barcelona, Spain

**Keywords:** *Streptococcus suis*, pig farm workers, meningitis, septic arthritis, Spain

## Abstract

**Introduction:** *Streptococcus suis (S. suis)* is a human zoonotic pathogen of occupational origin, with infection acquired through contact with live pigs or pig meat. Pig farming is one of Catalonia's biggest industries and as a result this region of Spain has one of the highest density pig populations per km^2^. The aim of our study was to describe the infections caused by *S. suis* occurring in that area over a 9-year period.

**Materials and Methods:** A retrospective, multi-center study was carried out by searching records from 15 hospitals in Catalonia for the period between 2010 and 2019.

**Results:** Over the study period altogether nine cases of *S. suis* infection were identified in five hospitals, with five of these cases occurring in the 2018–2019 period. The mean age of patients was 48 ± 8.9 years and all of them were males. Five patients (55.6%) worked in pig farms. The most frequent manifestation of infection was meningitis (5 cases; 55.6%) followed by septic arthritis (3 cases; 33.3%). None of the patients died at 30 days; nonetheless, 4 developed hearing loss as a long-term complication.

**Conclusion:** The most commonly identified *S. suis* infection was meningitis. Over 50% of the episodes occurred in the last 2 years and have affected pig farm workers. Further surveillance is needed in order to know its prevalence.

## Introduction

*Streptococcus suis* (*S. suis*) is a primary commensal of pigs, commonly colonizing the tonsils and nasal cavities of these animals (1). This bacterium can also infect other mammals such as wild boar and birds (2).

It is considered a human zoonotic pathogen of mainly occupational origin and was first reported in Denmark in 1968 (3). *S. suis* is classified into 35 serotypes with serotype two being the most virulent. It is frequently isolated both in swine and in humans (4).

Occupational exposition is a frequent antecedent in human infection. There are 5 major groups of workers exposed to *S. suis* infection: pig farmers, slaughterhouse workers, butchers and meat handlers, veterinarians and wild boar hunters (1). Other factors such as alcoholism, corticosteroid or immunosuppressive therapy, diabetes, splenectomy and gastrointestinal tumors may increase the risk of human infection (5).

**Figure 1:**
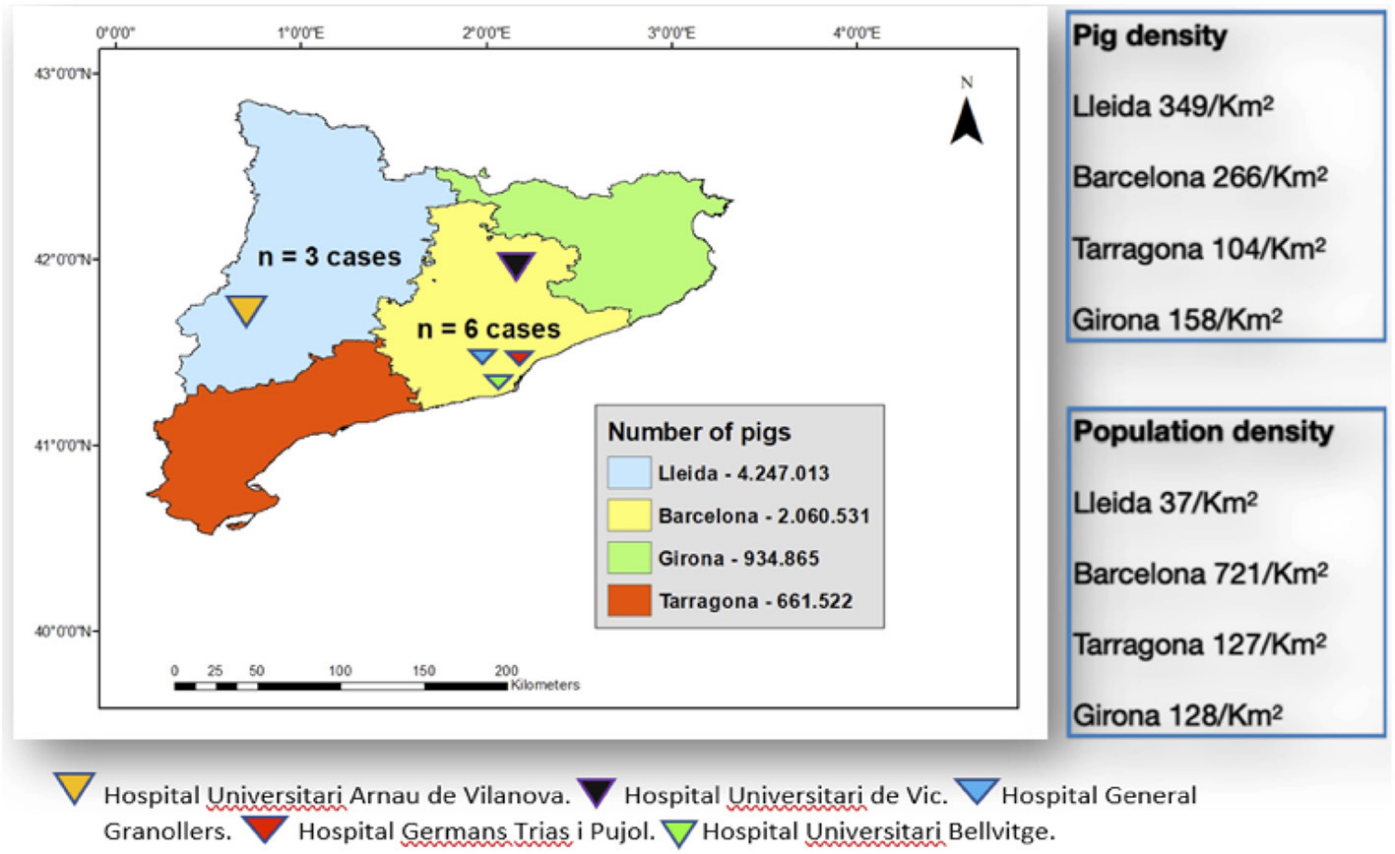
Map of the autonomous region of Catalonia in Spain showing the number of *S. suis* invasive infections, the density of pigs/km^2^ and the population/km^2^ by province.

Catalonia is the region of Spain with the biggest pig industry and thus represents one of the highest density pig populations per km^2^ in the country. Of the four provinces making up the Autonomous Region of Catalonia (Lleida, Barcelona, Tarragona and Girona), Barcelona (including the Osona region, with around 1,000 pigs/km^2^) (6) and Lleida are the two provinces with the highest density of pigs per km^2^ (Figure 1) (7).

Given this high density, it was felt of interest to examine the frequency and characteristics of *S. suis* invasive infections in humans in this geographic area.

## Materials and Methods

A retrospective, observational multi-center study of invasive *S. suis* infections was carried out by examining hospital records covering the period between January 2010 and December 2019. Of the 15 Catalan hospitals participating in the study, 11 were located in the province of Barcelona, three in the province of Girona and one in the province of Lleida. The epidemiological and clinical characteristics of all patients reported to have been infected by *S. suis* were retrospectively recorded in a database.

Invasive *S. suis* infection was defined by the isolation of *S. suis* from a normally sterile site (blood, cerebrospinal fluid, synovial fluid, seminal fluid) in a patient with signs and symptoms of infection.

Two *S. suis* infections previously described as sporadic cases (2), were included in this analysis.

The study was approved by the Ethics Committee of the Hospital Universitari de Vic; need for informed consent was waived (No. 2021163).

### Bacterial Identification and Antibiotic Susceptibility Testing

The identification of *S. suis* isolates was done using either the Vitek-2 (Biomèrieux^®^) or the MALDI-TOF (Bruker Daltonics) commercial system. The antimicrobial susceptibility test was performed through microdilution using commercially available Vitek-2 (Biomèrieux^®^) or MicroScan (Beckman) systems. The results were interpreted according to by the European Committee on Antimicrobial Susceptibility Testing (EUCAST) criteria (8).

### Statistical Analysis

For the qualitative variables, the percentages for each of the different categories were calculated. For the quantitative variables, the mean, median and standard deviation were calculated. The IBM^®^ SPSS^®^ Statistics programme version 26 was used to perform the statistical analysis.

## Results

**Figure 2:**
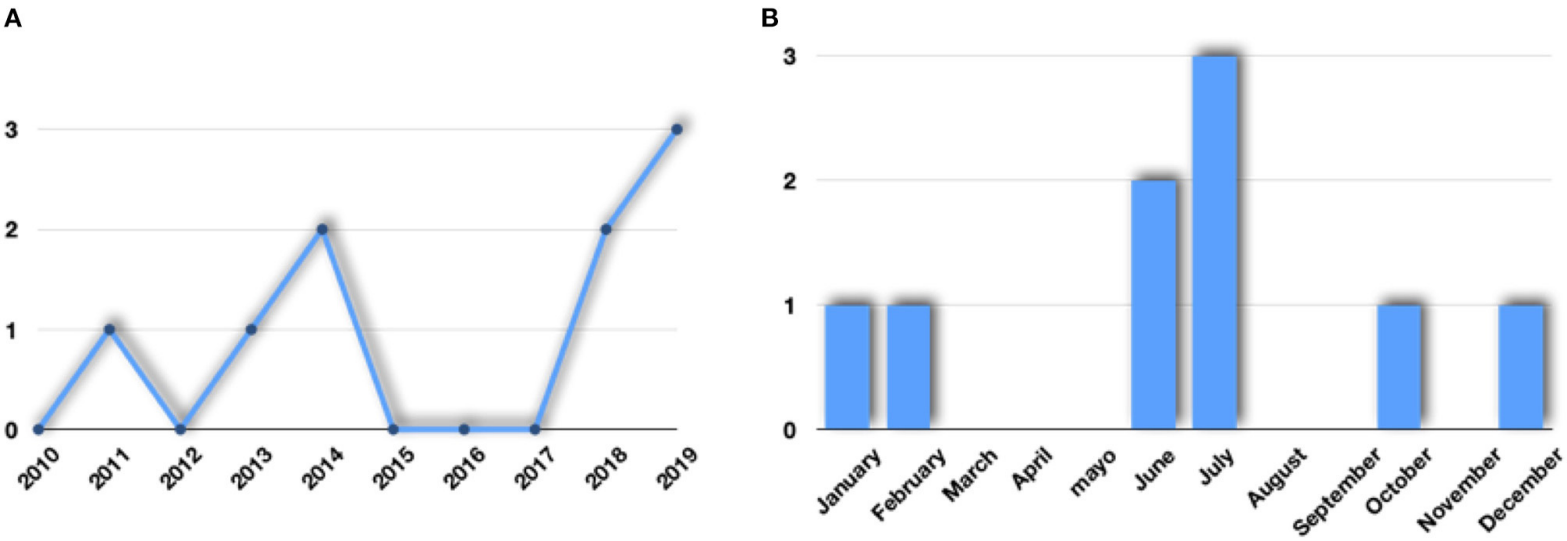
Distribution of cases of *S. suis* infections reported in Catalonia 2010–2019 by year **(A)** and by month **(B)**.

Nine cases of *S. suis* infections were detected at five hospitals, of which four were in the province of Barcelona and one in the province of Lleida. Of these nine cases, four (44.5%) were detected in the 2011–2014 period and five (55.5%) in the 2018-2019 period (Figure 2A).

Likewise, five cases occurred in summer while the remaining four cases occurred in autumn and winter (Figure 2B).

The mean age of the nine patients was 48 ± 8.9 years and the patients were all male. Charlson index scores were 0 (55.6%), 1 (22.2%), and 2 (22.2%). None of the patients were under corticosteroid or other immunosuppressive treatment at the time of infection.

Five patients (55.5%) worked in pig farms and only one patient had a pet (a cat). Four patients (44.4%) had handled meat in the previous week; one of them being a wild boar hunter who had suffered a wild boar bite and another was a butcher.

The most frequent manifestation of infection was meningitis (5; 55.5%), followed by septic arthritis (3; 33.3%) and there was one case each of native mitral valve endocarditis complicated by an ischemic stroke, periprosthetic infection of total hip prosthesis and orchitis.

The most frequent symptoms were fever (8 cases; 88.8%) and headache (5 cases; 55.5%). Three patients (33.3%) presented with septic shock.

None of the patients died at 30 days; nonetheless, four patients developed hearing loss as a long-term complication (one of them also developed ataxia).

Table 1 shows the clinical and epidemiological characteristics of each case, as well as the antibiotic treatment and the outcome.

**Table 1:** Clinical and epidemiological characteristics of cases of *S. suis* Infection.

	**Case 1**	**Case 2**	**Case 3**	**Case 4**	**Case 5**	**Case 6**	**Case 7**	**Case 8**	**Case 9**
Age	57	48	47	48	63	50	42	63	37
Sex	Male	Male	Male	Male	Male	Male	Male	Male	Male
Farm worker	No	No	No	Yes	Yes	No	Yes	Yes	Yes
Handled meat in previous week	Not known	Yes	Yes (butcher)	Yes	Not known	No	Yes	Not known	No
Pets	Cat	Not known	Not known	No	Not known	No	No	Not known	No
Underlying condition	Alcoholism	No	No	Hypertension, Glomerulonephritis	No	No	No	Alcoholism	Prosthetic hip
Charlson Index	1	0	0	0	2	1	0	2	0
Immunosuppressive treatment	No	No	No	No	No	No	No	No	No
Symptoms	Fever, headache	Fever, headache	Fever, headache	Fever, headache, encephalitis	Fever	Haematospermia	Fever, headache	Fever	Fever
Type of infection	Meningitis, septic arthritis	Meningitis, septic arthritis	Meningitis	Meningitis	Native mitral valve endocarditis	Orchitis	Meningitis	Septic arthritis	Prosthesis hip infection
Diagnostic sample	Blood culture, CSF	Blood culture, CSF	Blood culture, CSF	CSF	Blood culture	Seminal fluid	CSF	Blood culture, synovial fluid	Synovial fluid
Skin lesion	No	Yes (wild boar bite)	No	No	No	No	Yes	No	Yes
Septic shock	Yes	Yes	No	Yes	No	No	No	No	No
Days ICU	16	15	No	8	No	No	No	No	No
Endotracheal intubation	Yes	Yes	No	Yes	No	No	No	No	No
Definitive antibiotic	Ceftriaxone	Ceftriaxone	Ceftriaxone	Penicillin	Penicillin	Erythromycin	Ceftriaxone	Ceftriaxone	Ceftriaxone + Clindamycin
Dexamethasone	Yes	Not known	Not known	No	No	No	Yes	No	No
Mortality	No	No	No	No	No	No	No	No	No
Sequelae	Hearing loss, ataxia	Hearing loss	No	Hearing loss	No	No	Hearing loss	No	No

*CSF, Cerebrospinal fluid; ICU, Intensive Care Unit*.

All of *S. suis* strains were susceptible to 3rd generation cephalosporins, vancomycin and rifampicin, 88.9% to penicillin and fluoroquinolones, respectively and 71.4% to trimethoprim-sulfamethoxazole. Most strains were resistant to erythromycin and clindamycin (both 88.9%). Table 2 shows the MICs of *S. suis* to the antibiotics tested.

**Table 2:** Antibiotic MICs of *Streptococcus suis*.

**Antimicrobials**	**Case 1**	**Case 2**	**Case 3**	**Case 4**	**Case 5**	**Case 6**	**Case 7**	**Case 8**	**Case 9**
Penicillin	<0.03	<0.03	0.06	<0.03	0.023	**0.5^c^**	<0.06	0.047	0.064
Cefotaxime	0.12	<0.06	0.12	<0.06	<0.03	<0.06	0.5	0.125	<0.06
Levofloxacin	0.5	0.5	0.5	0.5	<0.25	**8**	<0.25	0.5	0.19
Erythromycin	**>4^b^**	**>4**	**>1**	**>4**	**>4**	<0.12	**>8**	**>0.5**	**>4**
Clindamycin	**>4**	**>4**	**>1**	**>0.5**	**>4**	**0.5**	**>1**	**>0.5**	<0.25
SXT^a^	**>2**	<0.5	<0.5	<0.5	**>2**	<0.5	<1	0.25	<0.5
Rifampicin	<0.25	<0.25	<0.5	<0.5	<0.5	<0.5	<0.06	<0.5	<0.5
Vancomycin	<0.5	<0.5	1	<0.5	0.25	0.5	0.25	0.5	0.5

a *SXT, trimethoprim-sulfamethoxazole*.

b *Numbers in boldface indicate resistance values according to EUCAST*.

c *Non-penicillin-susceptible*.

Five patients were treated with ceftriaxone, one patient with ceftriaxone plus clindamycin, two patients with penicillin and one patient with erythromycin. The mean duration of treatment was 25.8 ± 25.5 days.

## Discussion

Despite being a leading pig producer in Europe, few *S. suis* infections have been reported in Spain (5). In this study we describe a series of cases of invasive *S. suis* infections in an area of high swine density in Spain.

In our series, invasive *S. suis* infections occurred more frequently in summer. These data coincide with those reported in Wangkaew et al. (9) and suggest that *S. suis* infections could be related to high ambient temperatures.

Although the number of cases is low, most of them occurred in the last 2 years suggesting surveillance of a putative increase in the future. However, it could be partially explained by *S. suis* misidentification in the past as reported by other authors (10).

Several studies have demonstrated that *S. suis* infection in humans is associated with occupational or environmental exposure to live pigs or pig meat (1). In fact, in a series study published in the Netherlands, 93% of those infected by *S. suis* were pig farmers, abattoir workers or butchers, or had occasional contact with pigs or pork products (11). Similarly, six (66.6%) of the patients in the present study were farmers or butchers, the remaining three being residents of areas with an unusually high density of pigs (Lleida and the Osona region of Barcelona), although they did not have any direct contact with farms.

*S. suis* infections have mostly been reported in Asian countries, especially Thailand, Vietnam and China (12). Infections described have included meningitis, infective endocarditis, sepsis syndrome, arthritis, pneumonia, endophthalmitis and spondylodiscitis (9). Recently Kerdsin et al. analyzed genotypic diversity in multiple *S suis* infections. The authors observed that in 83.7% of meningitis cases the clonal complex CC1 was isolated followed by CC104 (14.9%), Sepsis could be associated with isolates belonging to all CCs and two singletons (ST235 and ST236). CC1 isolates also accounted for 47.7% of the sepsis cases, followed by those of CC104 (37.3%). Infective endocarditis was mainly caused by CC1 isolates. Septic arthritis cases were caused by CC1 isolates, whereas CC104 and CC233/379 isolates were associated with spontaneous peritonitis. Serotype two (94.6%) was predominant, followed by serotype 14 (4.5%) (13). In Europe, the Netherlands is one of the countries with the highest density of pigs, and cases of meningitis in humans caused by *S. suis* have been described (11). In our series meningitis was the most commonly of *S. suis* infection and was frequently complicated by long-term hearing loss. Hearing loss occurred in 80% of our patients, similar to the rate described in patients with *S. suis* meningitis in the Netherlands (86%) (11), although higher than data reported for Vietnam (50%) (12). The role of dexamethasone in preventing hearing loss after *S. suis* meningitis is controversial (11, 14).

It should be noted that a patient with haematospermia was diagnosed with orchitis. In this particular case, neither contact with animals nor meat consumption nor any other risk factor for *S. suis* infection was detected, although the patient lived in an area with a high density of pig farms.

Mortality from invasive *S. suis* infections is high. Eighty-four out of the 659 cases with *S. suis* infection diagnosed in Thailand were fatal (12.7%), of which 21 cases were attributed to meningitis, 62 cases to sepsis, and one case was to infective endocarditis (13). As we have noted, none of the patients in our series died, and all the patients with meningitis survived. Compared to other causes of meningitis in an age-matched population, the number of lethal cases of *S. suis* meningitis is relatively low (15). This may be due to the fact that the majority of people infected with *S. suis* are healthy adults with a low comorbidity score.

Early detection of human *S. suis* infection is crucial, as rapid progression of symptoms, such as sepsis or septic shock, is one of the risk factors for high mortality (16, 17). However, traditional diagnostic methods are routine and complex, and sometimes fail to adequately diagnose the infection. Several molecular biological techniques have been developed for diagnosis, but they are not suitable for small hospitals or for field diagnostics. Nakayama et al. (18) developed the immunochromatographic banding (ICS) technique against *S. suis* serotype 2 to detect *S. suis* antigens in urine and could be a good option for rapid diagnosis.

Antimicrobial resistance is a major health problem; data suggest an increasing and alarming contribution of *S. suis* to this global threat (19). Bamphensin et al. (20) analyzed 448 *S. suis* isolates recovered from human infections in Thailand and characterized them for their antimicrobial susceptibility. A total of 37 (8.2%) showed intermediate resistance to penicillin. Isolates belonging to serotype 2 ST233 were the most common strain type associated with penicillin non-susceptibility. Serotype 14 (ST105) and serotype 24 (ST221 and ST234) were also found to be intermediately resistant to penicillin. In our study, one (11%) patient had an infection due to penicillin-non-susceptible *S. suis* in 2019. Although just one case out of nine, we must be aware of an eventual increase of cases caused by *S suis* penicillin non-susceptibility strains in the future in Catalonia, Spain.

The present study has several limitations, mainly due to its retrospective design. The unavailability of serotypes and the shortness of the series make it difficult to draw significant conclusions.

In summary, invasive *S. suis* infections are rare in Spain, although there appears to have been an increase in recent years. While most of the patients had a history of contact with live pigs or pig meat, this was not always the case. Meningitis was the most prevalent clinical manifestation and hearing loss was a common and serious complication.

## Study Group For *S. suis*

Rafel Perez Vidal (Hospital de Sant Joan de Déu, Manresa), Lorena Gaviria (Hospital de Cerdanya, Puigcerdà), Goretti Sauca-Subias (Hospital de Mataró), Carme Gallès (Hospital de Blanes), Josefa Perez (CATLAB Hospital de Terrassa and Hospital Mutua de Terrassa), Oriol Gasch (Hospital Universitari Parc Taulí, Sabadell), Alex Soriano (Hospital Clinic de Barcelona, Barcelona) Vicents Brito (Hospital Sant Boi de Llobregat), Nieves Larrosa (Hospital Universitari Vall d'Hebron, Barcelona).

## Data Availability Statement

Requests to access the data analyzed in this study should be directed to Esteban Reynaga, eareynaga.germanstrias@gencat.cat.

## Author Contributions

All authors listed in the contributors' affiliations meet the ICMJE Authorship Criteria, that is, they contributed substantially to the study's conception and design, the acquisition of data, the drafting of the article, its critical review, and the final approval of the manuscript.

## Conflict of Interest

The authors declare that the research was conducted in the absence of any commercial or financial relationships that could be construed as a potential conflict of interest.

## Publisher's Note

All claims expressed in this article are solely those of the authors and do not necessarily represent those of their affiliated organizations, or those of the publisher, the editors and the reviewers. Any product that may be evaluated in this article, or claim that may be made by its manufacturer, is not guaranteed or endorsed by the publisher.
