# Supplementary material for: Clinical and Epidemiological Characteristics of Streptococcus suis Infections in Catalonia, Spain
Source: Front Med (Lausanne). 2021 Dec 8;8:792233. doi: 10.3389/fmed.2021.792233 (PMC8692758; doi:10.3389/fmed.2021.792233)
Supplement: Supplementary file 1 [file Data_Sheet_1.docx]

**SUPPLEMENTARY DATA**

^11^ Study group for *S suis*: Rafel Perez Vidal (Hospital de Sant Joan de Déu, Manresa), Lorena Gaviria (Hospital de Cerdanya, Puigcerdà), Goretti Sauca-Subias (Hospital de Mataró), Carme Gallès (Hospital de Blanes), Josefa Perez (CATLAB Hospital de Terrassa and Hospital Mutua de Terrassa), Oriol Gasch (Hospital Universitari Parc Taulí, Sabadell), Alex Soriano (Hospital Clinic de Barcelona, Barcelona) Vicents Brito (Hospital Sant Boi de Llobregat), Nieves Larrosa (Hospital Universitari Vall d’Hebron, Barcelona)
